# Gating control and K^+^ uptake by the KAT1 K^+^ channel leaveraged through membrane anchoring of the trafficking protein SYP121

**DOI:** 10.1111/pce.13392

**Published:** 2018-08-01

**Authors:** Cécile Lefoulon, Sakharam Waghmare, Rucha Karnik, Michael R. Blatt

**Affiliations:** ^1^ Laboratory of Plant Physiology and Biophysics, Bower Building University of Glasgow Glasgow UK

**Keywords:** inward‐rectifier/single‐channel analysis/binding, Qa‐SNARE/K^+^ channel, voltage‐dependent/protein conformation/plant cell expansion

## Abstract

Vesicle traffic is tightly coordinated with ion transport for plant cell expansion through physical interactions between subsets of vesicle‐trafficking (so‐called SNARE) proteins and plasma membrane Kv channels, including the archetypal inward‐rectifying K^+^ channel, KAT1 of *Arabidopsis*. Ion channels open and close rapidly over milliseconds, whereas vesicle fusion events require many seconds. Binding has been mapped to conserved motifs of both the Kv channels and the SNAREs, but knowledge of the temporal kinetics of their interactions, especially as it might relate to channel gating and its coordination with vesicle fusion remains unclear. Here, we report that the SNARE SYP121 promotes KAT1 gating through a persistent interaction that alters the stability of the channel, both in its open and closed states. We show, too, that SYP121 action on the channel open state requires SNARE anchoring in the plasma membrane. Our findings indicate that SNARE binding confers a conformational bias that encompasses the microscopic kinetics of channel gating, with leverage applied through the SNARE anchor in favour of the open channel.

## Abstract 2

Plant cells maintain turgor pressure against the cell wall. So vesicle traffic and solute uptake must be tightly coordinated during cell expansion. Secretory vesicle traffic and K^+^ channel activities are promoted by physical interactions between subsets of vesicle‐trafficking proteins and Kv channels at the plant plasma membrane, but the temporal relationship between these processes has remained unclear. Ion channels open and close rapidly over milliseconds, whereas vesicle fusion requires periods of many seconds. Here, we show that the SNARE binding effects long‐term changes in channel gating kinetics through conformations of the channel voltage sensor that depend, in part, on the leverage afforded by anchoring of the SNARE in the plasma membrane.

## 1 INTRODUCTION

Plant cells use the potassium ion (K^+^) to maintain hydrostatic (turgor) pressure that drives cell expansion, growth, and facilitates changes in guard cell volume for stomatal movements (Jezek & Blatt, 2017; Ward, Maser, & Schroeder, 2009). Potassium uptake and distribution within the plant relies on both high‐affinity, H^+^‐coupled K^+^ transporters and K^+^ channels mediating K^+^ ion flux across cell membranes, with uptake by K^+^ channels responsible for roughly 50% of the total K^+^ content of the plant under most field conditions (Amtmann & Blatt, 2009; Rubio, Nieves‐Cordones, Aleman, & Martinez, 2008). These channels define much of the conductance characteristics of virtually every tissue. Their regulation is therefore of vital relevance to the balance of K^+^ flux in every aspect of the cellular physiology of plants.

The KAT1 channel of *Arabidopsis* is the archetypal inward‐rectifying K^+^ channel of the plant plasma membrane and shares a number of structural features with the voltage‐sensitive Kv superfamily of K^+^ channels of eukaryotes (Dreyer & Blatt, 2009; Palovcak, Delemotte, Klein, & Carnevale, 2014). Kv channels assemble as tetramers, each subunit consisting of six transmembrane α‐helices. The first four α‐helices (S1‐S4) of the channel protein form a semi‐autonomous voltage‐sensor domain (VSD) structure. These VSDs incorporate a series of fixed positive charges that, as membrane voltage changes, drive VSD conformation to move it within the membrane and draw open the channel pore (Dreyer & Blatt, 2009; Labro, Lacroix, Villalba‐Galea, Snyders, & Bezanilla, 2012; Lai, Grabe, Jan, & Jan, 2005; Lefoulon et al., 2014). VSD conformation thus serves the dual function of sensor and regulator for voltage control of K^+^ flux, connecting channel activity to that of other transporters in the membrane, especially to the H^+^‐ATPase and its role in membrane energization.

KAT1 and KC1, a close channel homolog, also bind directly and selectively with the plasma membrane protein SYP121 through a conserved RYxxWE motif located at the cytosolic surface of the VSD (Grefen et al., 2015; Honsbein, Blatt, & Grefen, 2011). SYP121 is one of two so‐called Qa‐SNAREs (Soluble NSF Attachment protein REceptor) proteins that facilitate accelerated vesicle traffic at the plasma membrane of *Arabidopsis* (Karnik et al., 2017). In general, SNAREs from target and vesicle membranes drive vesicle fusion and membrane intercalation by assembling in ternary complexes of cognate partner Qa‐, Qb‐, Qc‐ and R‐SNAREs, designations defined by the respective residues at the core of the SNARE complex (Lipka, Kwon, & Panstruga, 2007). SYP121 binding with the K^+^ channels is independent of channel traffic, however. Instead, SYP121 binds with the channels that are already located at the plasma membrane, and the interaction regulates SYP121‐mediated vesicle traffic, enhancing vesicle fusion to facilitate secretion and membrane expansion (Grefen et al., 2015). SYP121 binding also moderates the activities of K^+^ channels at the plasma membrane to promote K^+^ uptake, thereby coordinating the rates of K^+^ uptake with secretory traffic for growth (Honsbein et al., 2009; Karnik et al., 2017). Indeed, vesicle traffic and solute transport must be tightly coordinated to maintain solute content and cell surface area during plant cell expansion. Such coupling between SNAREs and channel VSDs may be common in plants. The binding motifs on both proteins are closely conserved within subsets of plasma membrane SNAREs and K^+^ channels in vascular plants, implying their co‐evolution as the number of SNARE genes expanded when plants colonized land (Karnik et al., 2017; Sanderfoot, 2007).

Clearly, the molecular mechanics of the SNARE‐channel interaction is particularly important. Not only can this knowledge inform on how channel activity is integrated with the SNARE complex assembly during vesicle traffic, but it is also relevant to understanding the process of channel gating and K^+^ uptake. In general, channel activity arises from millisecond transitions between open and closed conformations of the channel pore. What is not obvious is how these events might be coordinated temporally with the very much slower process of SNARE‐mediated vesicle fusion (Jahn & Scheller, 2006). To address this question, we have made use of heterologous expression in *Xenopus* oocytes which ensures an environment uncomplicated by other, native plant proteins in which to study the SNARE‐channel interactions in isolation. Here, we report that SYP121 binding to KAT1 alters the stability of the channel “open” and “closed” states to promote channel activity. Additionally, we show that the bias introduced by binding depends on membrane anchoring of SYP121. Thus, the SNARE alters KAT1 activity through long‐lived alterations to the conformational stability of its VSD. These findings suggest, too, that the bound channel may be integrated stably within the SNARE complex assembly during vesicle fusion.

## 2 METHODS

### 2.1 Molecular biology, oocyte expression, and recombinant protein purification

KAT1, KAT1^W62A^, SYP121, and SYP121^ΔC^ coding sequences were subcloned and transferred in pGT‐Dest and pGT‐Dest‐myc by LR reaction as described previously (Grefen et al., 2010;Grefen et al., 2015 ; Lefoulon et al., 2014). cRNA was transcribed after template linearization using T7 mMessage mMachine (Ambion, USA) and cRNA quality confirmed by gel electrophoresis. cRNA was injected into stage VI *Xenopus* oocytes as before (Grefen et al., 2015; Lefoulon et al., 2014) in ratios as indicated, and patch and voltage clamp measurements were carried out 3‐ to 5‐days post‐injection.

For patch clamp studies, the vitelline membrane was first removed with forceps after exposing oocytes to a hypertonic solution of the standard buffer plus 245 mM sucrose. SYP121^ΔC^ was expressed and purified from Escherichia coli BL21 DE3 cells (Karnik et al., 2013) prior to transfer to the final recording buffer.

### 2.2 Electrophysiology and immunochemistry

Whole‐cell currents were recorded under voltage clamp using an Axoclamp 2B amplifier (Axon Instruments, Foster City, CA; Grefen et al., 2015; Lefoulon et al., 2014; Leyman, Geelen, Quintero, & Blatt, 1999). Measurements were performed under continuous perfusion with 30 mM KCl and 70 mM NaCl with 1 mM CaCl_2_, 1.5 mM MgCl_2_, and 10 mM HEPES‐NaOH, pH 7.3. Recordings were analyzed using Henry IV software (Y‐Science, Glasgow UK), as described previously (Grefen et al., 2015; Honsbein et al., 2009; Lefoulon et al., 2014). Patch clamp recording were performed on inside–out membrane patches. Borosilicate pipettes with input resistances of 7–10 MΩ were pulled with a PP‐83 puller (Narishige, Tokyo). Patch and macropatch recordings were recorded with an Axopatch 200B amplifier driven by Heka software (Heka Elektronik, Lambrecht). Currents were recorded in symmetrical 135 mM K^+^‐gluconate, with 4 mM MgCl_2_, 3 mM EGTA, 0.5 mM CaCl_2_, and 7 mM HEPES‐NaOH, pH 7.2, at the outer face of the membrane and with 2 mM CaCl_2_, 6 mM MgCl_2_, and 10 mM HEPES‐NaOH, pH 7.2, to give 70 nM free [Ca^2+^] at the inner face of the membrane (Schoenmakers, Visser, Flik, & Theuvenet, 1992). SYP121^ΔC^ was added to the bath at 1 μM unless otherwise noted. Single‐channel currents were filtered at 1 or 3 kHz with an 8‐pole Bessel filter and recorded at 40 kHz. Data segments were collected offline with Acquire (Bruxton, Seattle, USA) and filtered digitally for recurrent noise with N‐PRO (Y‐Science, Glasgow UK). Single‐channel events were reconstructed using the Hidden Markov Model approach with Bayesian restoration (Fredkin & Rice, 1992) of PANDORA! (YScience) and lifetime distributions determined using TAC and TACfit (Bruxton).

Oocytes were collected after whole‐cell recording, homogenized in denaturing buffer (150 mM NaCl, 0.25% SDS, 1% NP‐40, 1 mM EDTA, 1 mM NaF, 1.125 mM DMFS, and 50 mM Tris–HCl, pH 7.4) at 10 μl/oocyte, and centrifuged at 425 g for 5 min. One volume of loading buffer (4 M Urea, 10% SDS, 40 mM EDTA, 0.2% Triton, 0.1% bromophenol blue, 20% Glycerol, 200 mM DTT, 100 mM Tris–HCl, pH 6.8) was added to the supernatant, and samples were incubated 30 min at 37°C before transfer to nitrocellulose. Protein were detected by Western Blot ECL Advance kit (GE Healthcare, Poole UK) with rabbit α‐myc (dilution 1:5000, Abcam, Cambridge UK) for KAT1 and KAT1^W62A^, α‐SYP121 antibodies (dilution of 1:4000) for SYP121 and SYP121^ΔC^ (Honsbein et al., 2009), and secondary horseradish peroxidase‐coupled goat, anti‐rabbit antibodies (Abcam).

### 2.3 Statistics

Data are reported as means ±SE of *n* independent experiments, with post‐analysis carried out by ANOVA or Student's *t* test. Joint non‐linear, least squares fittings were carried out using the Marquardt–Levenberg algorthm of SigmaPlot v.11 (SPSS, Poole UK).

## 3 RESULTS

### 3.1 SYP121 alters the voltage sensitivity of KAT1

**Figure 1:**
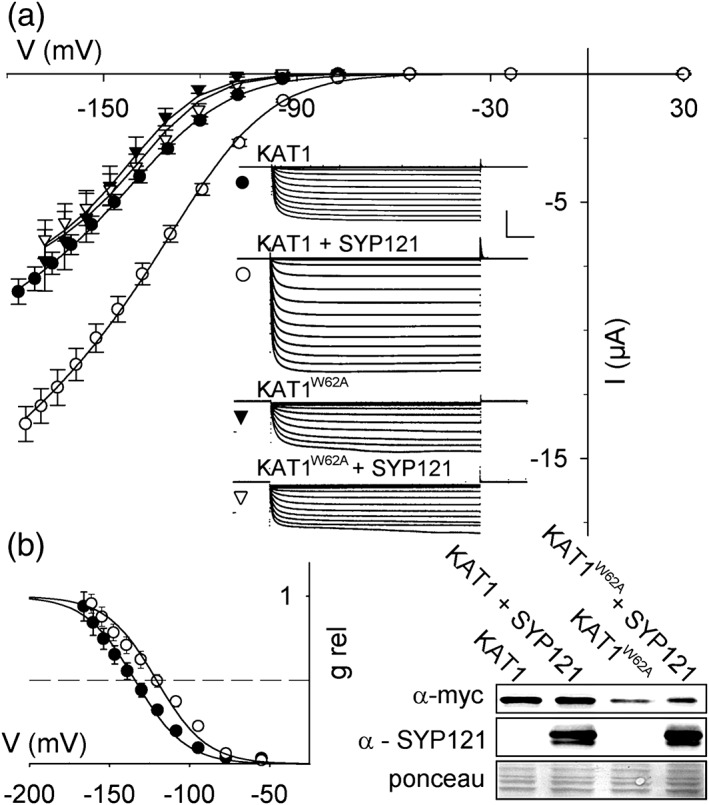
SYP121 enhances whole‐cell KAT1 current. (a) Steady‐state current–voltage curves (means ±*SE*, *n* > 5 for each data set) and representative current traces recorded on expressing KAT1 (circles) and the non‐interacting KAT1^W62A^ mutant (triangles) without (filled symbols) and with SYP121 (open symbols) using a 1:4 molar ratio of cRNAs (Grefen et al., 2015; Honsbein et al., 2009). Voltage was stepped from a holding voltage of −20 mV to voltages from +30 mV to −180 mV. Solid curves are the results of joint, least squares fitting to the Boltzmann function
(3)$I_{\text{KAT1}}=g_{\max}\;\left(V-E_{K}\right)/\left(1+{{e^{\delta (V-V}}_{1/2}}^{)F/\mathrm{RT}}\right),$where g_max_ is the conductance maximum, E_K_ is the equilibrium voltage for K^+^, V_1/2_ is the voltage at mid‐point for maximal conductance; δ is the apparent gating charge or voltage sensitivity coefficient. F, R, and T have their usual meaning. Mean g_max_ increased from 0.061 ± 0.009 to 0.096 ± 0.006 mS, and V_1/2_ shifted from −132 ± 1 mV to −119 ± 1 mV with SYP121 (*P* < 0.001). KAT1^W62A^ current shown scaled (multiplied) by 4 for comparison; its parameters were unaffected by SYP121 (g_max_, 0.013 ± 0.002 mS; V_1/2_–136 ± 2 mV). *Insets*: Representative current traces cross‐referenced by symbol. Scale: 5 μA (vertical), 1 s (horizontal). (b) Relative conductance‐voltage curves for KAT1 ± SYP121 from (a). Symbols are corresponding tail current amplitudes scaled with the midpoint to give 0.5. Solid line is the fitting of Boltzmann function derived as g/g_max_ Equation (3). Dashed line marks 0.5 g_max_. Immunoblot analysis of myc‐tagged KAT1 and SYP121 from representative oocytes collected after recordings. Ponceau is included as a loading control

We expressed KAT1 alone and together with SYP121 in *Xenopus* oocytes to compare the channel activities in isolation from the plant. Initially, channel current was recorded by two‐electrode voltage clamp for analysis of the ensemble current. Steps to −100 mV and more negative voltages yielded inward current relaxations that approached a new steady state within 500 ms (Figure 1a), and steady‐state current–voltage (IV) analysis showed a clear inward rectification, much as has been described previously (Lefoulon et al., 2014). By contrast, oocytes injected with water or with SYP121 only showed no appreciable current under these same conditions (less than 0.3 nA inward current at −140 mV, not shown; see Grefen, Donald, et al. [2010], Grefen, Karnik, et al. (2015), and Honsbein et al., 2009). Co‐expression with SYP121 enhanced the KAT1 current amplitude and displaced the IV curve to more positive voltages. Binding of SYP121 is disrupted by mutating the R^58^YxxWE motif of KAT1 (Grefen et al., 2015). To confirm that the effects of SYP121 expression depended its interaction with KAT1, we co‐expressed SYP121 with the KAT1^W62A^ mutant. The amplitude of the current, both with and without SYP121, was reduced in this mutant, consistent with its lower expression (Figure 1b). However, the KAT1^W62A^ current was unaffected by co‐expression with SYP121, confirming the requirement for SYP121 binding to promote channel gating.

To estimate the effects of SYP121 on KAT1 gating, we fitted steady‐state IV curves to a Boltzmann function (Figure 1, Equation (3)) to extract the macroscopic conductance, g_max_, and the gating characteristics defined by the apparent gating charge, δ, and the voltage yielding half‐maximal conductance, V_1/2_. g_max_ is affected by the number of channels at the membrane that are active; δ and V_1/2_ reflect the intrinsic voltage sensitivity and free energy of channel gating, and so give insight into how the channels are regulated by voltage. SYP121 expression led to almost a twofold increase in g_max_ and a +10 mV shift in V_1/2_, without an apparent change in δ. We validated these findings by recording tail current amplitudes at +20 mV following channel activation at each voltage (Figure S1). Scaling the relative amplitudes against those determined at the previously estimated values for V_1/2_ gave voltage dependencies that were indistinguishable from relative conductances derived from the Boltzmann fittings (Figure 1b). Thus, like KC1 (Honsbein et al., 2009), co‐expression of SYP121 with KAT1 has an appreciable effect on the voltage sensitivity of the channel gate as well as its macroscopic conductance.

**Figure 2:**
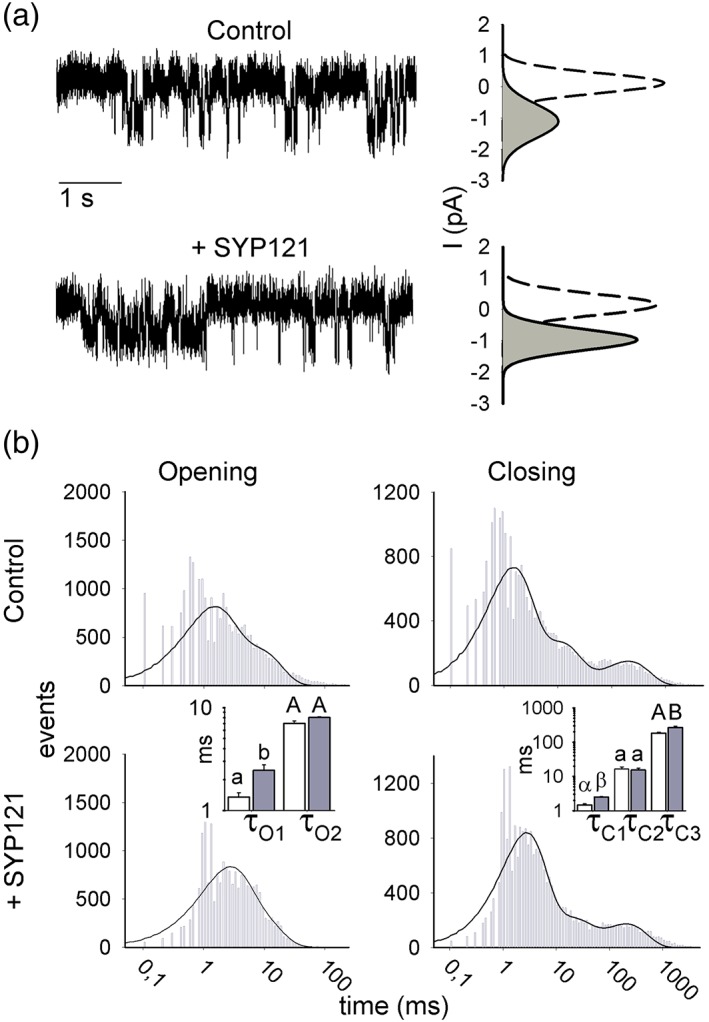
SYP121 alters both open and closed lifetimes of KAT1. (a) Representative single‐channel currents from inside–out patches recorded at −120 mV for KAT1 alone (*above*) and with SYP121 (*below*) with fitted amplitude histograms for the open (*solid lines, grey fill*) and close state (*dashed lines*). Open‐state curves are scaled (multiplied) by 4 relative to the closed state for clarity. Amplitudes: 1.06 ± 0.21 pA (KAT1), 1.05 ± 0.11 pA (KAT1 + SYP121). (b) Frequency histograms of KAT1 open and close durations for KAT1 alone (*above*) and with SYP121 (*below*) plotted on logarithmic scales. Solid lines are fittings to Equation (2) to extract the dwell time constants. Bins below 1 ms (Nyquist limit) are shown but were not included in fittings. Open and closed lifetimes were fitted satisfactorily with sums of two and three exponentials, respectively. Mean dwell time constants ±*SE* (*n* = 4) for open and closed lifetimes are summarized by the bar charts (KAT1, open bars; KAT1 + SYP121, filled bars). Significant differences ±SYP121 are indicated by lettering (see Table 1) [Colour figure can be viewed at http://wileyonlinelibrary.com]

We used patch clamp methods to resolve single‐channel currents of KAT1 expressed in the oocytes and thereby identify the microscopic properties of channel gating affected by SYP121. KAT1 channels were identified initially on the basis of their inward rectification and single‐channel conductance, around 6–9 pS (see Jezek & Blatt, 2017 and references therein), which is an order of magnitude smaller than the dominant Ca^2+^‐activated Cl^−^ channels that contribute to the so‐called leak current of the oocytes (Sigel, 1990; Weber, Liebold, Reifarth, & Clauss, 1995). Single KAT1 channels showed amplitudes—and hence, a single‐channel conductance—that were unaffected (*P* = 0.96) by expression together with SYP121 (Figure 2a); however, co‐expression with SYP121 enhanced the mean open probability, P_o_ (*P* < 0.02), which reflects the fraction of time in which the channels are open. Calculated as the fractional peak amplitude compared with that of the closed channel, P_o_ values were 5.5 ± 1.1% and 10.3 ± 1.3% for KAT1 alone and when co‐expressed with SYP121, respectively. Statistically indistinguishable results were obtained using the fractional opening time from steady‐state recordings of 2‐ to 3‐min duration (P_o_ = 5.3 ± 0.6% [KAT1], 9.3 ± 1.7% [KAT1 + SYP121]).

The free energy change in gating imposed by SYP121, ΔΔG, can be calculated from the ratio of open and closed probabilities, P_o_ and P_c_ respectively, as well as from the shift in the steady‐state IV curve as follows:

(1ab) $$\mathrm{\Delta \Delta G}=-\mathrm{RT}\;\left(\ln\;\left[P_{o,+\mathrm{SYP}}/P_{c,+\mathrm{SYP}}\right]-\ln\;\left[P_{o,-\mathrm{SYP}}/P_{c,-\mathrm{SYP}}\right]\right)=-\mathrm{F\delta}\;\left(V_{1/2,+\mathrm{SYP}}-V_{1/2,-\mathrm{SYP}}\right),$$

where the subscripts ±SYP indicate with and without SYP121, and R and T have their usual meanings. Analysis of the data in Figures 1 and 2a from whole‐cell and from single‐channel recordings yielded similar ΔΔG values of −0.37 ± 0.18 kcal/mol and −0.35 ± 0.12 kcal/mol, respectively. These values show that the free‐energy changes estimated from the open and closed probabilities and from the shift in V_1/2_ in whole‐cell recordings with SYP121 are indistinguishable, supporting the identity of the KAT1 single‐channel currents and the conclusion of a common effect of SYP121 mediated through the free energy of channel gating.

### 3.2 SYP121 alters KAT1 open and closed lifetimes

The studies outlined above demonstrate the time‐averaged effects of the SNARE‐channel interactions, but they do not resolve their underlying dynamics. We wanted to know if SYP121 binding/debinding was sufficiently rapid to “trap” the channel in the open state. To resolve the microscopic kinetics of SYP121 action, we made use of single‐channel recording to extract the open and closed lifetimes for KAT1. The temporal kinetics of channel gating are evident as rapid (typically millisecond) transitions in current between the closed and open states of a channel and the dwell times in each state. For Kv channels, these transitions are coupled to VSD conformation (Dreyer & Blatt, 2009; Palovcak et al., 2014). Thus, the voltage‐dependence of KAT1 and other plant Kv channels arise from changes in the dwell times in each state and so‐called “bursts” in activity that reflect substate transitions of the voltage sensor with increasing voltage (Zei & Aldrich, 1998).

We plotted the dwell times in the open and closed states as frequency histograms from experiments with inside–out patches. Rundown was observed in only a small number of experiments, and these records were omitted from analysis (Zei & Aldrich, 1998). Transitions were analysed from four independent experiments each expressing KAT1 alone and with SYP121. Satisfactory fitting were obtained with a sum of two exponentials for the open and three for closed lifetimes (Figure 2b) using the frequency distribution equation:

(2) $$f\;\left(t\right)={\Sigma \omega }_{i}a_{i}\;{\tau_{i}}^{-1}e^{-t/\tau_{i}},$$

consistent with the major lifetime distributions previously reported for KAT1 (Zei & Aldrich, 1998). Here, a_i_ is the component amplitude fraction such that Σa_i_ = 1, τ_i_ is the component dwell time constant, and t is the dwell time interval. To accommodate frequency distributions across several orders of magnitude, Equation (2) incorporates the weighting factor ω_i_, corresponding to the length of each bin multiplied by the sum of events within the bin. Each peak thus defines the corresponding dwell time constant. We found no significant effect on the fractional component amplitudes (Table S1), but co‐expression with SYP121 led to very significant changes in both open and closed lifetimes for KAT1. Notably, τ_O1_ for the shortest open lifetimes increased substantially with SYP121. Additionally, τ_C1_ and τ_C3_, corresponding to the shortest and longest‐lived closed lifetimes, were also affected (Figure 2b and Table 1). Thus, the effect of SYP121 co‐expression on KAT1 transitions is distributed between closed and open states, with the overall effect favouring a longer dwell time in the channel open state.

**Table 1:** Lifetime analysis for KAT1 and the actions of co‐expression with SYP121 and of SYP121^ΔC^ additions^a^

	Open lifetime		Closed lifetime		
*SYP121* (Figure 2)	τ_O1_	τ_O2_	τ_C1_	τ_C2_	τ_C3_
KAT1	1.31 ± 0.10^A^	7.04 ± 0.42	1.48 ± 0.14^a^	16.2 ± 2.8	183 ± 11^A^
KAT1 + SYP121	2.54 ± 0.23^B^	8.04 ± 0.13	2.56 ± 0.16^b^	15.4 ± 2.0	265 ± 28^B^

	Open lifetime		Closed lifetime		
*SYP121* ^Δ*C*^ (Figure 3)	τ_O1_	τ_O2_	τ_C1_	τ_C2_	τ_C3_
KAT1	1.59 ± 0.23	8.56 ± 0.94	1.79 ± 0.35	22.3 ± 5.3	168 ± 18^A^
KAT1 + SYP121^ΔC^	1.87 ± 0.30	10.14 ± 1.7	1.76 ± 0.27	21.4 ± 3.6	301 ± 24^B^

	Closing lifetime
*SYP121* ^Δ*C*^ (Figure 4, [1])	τ_C3_
KAT1	168 ± 17^A^
KAT1 + SYP121^ΔC^	301 ± 24^B^
Washout −120 mV	285 ± 13^B^

	Closing lifetime
*SYP121* ^Δ*C*^ (Figure 4, [2])	τ_C3_
KAT1	178 ± 17^A^
KAT1 + SYP121^ΔC^	301 ± 27^B^
Washout 0 mV	404 ± 98^B^

	Closing lifetime
*SYP121* ^Δ*C*^ (Figure 4, [3])	τ_C3_
KAT1	183 ± 23
SYP121 and washout 0 mV	213 ± 48

a Time constants are as reported in Figures 2, 3, and 4 and are indicated within each subtable. For Figure 4, the protocol is referenced in square brackets. Lower case letters indicate significant differences for any one time constant at *P* < 0.01. Upper case letters indicate significant differences for any one time constant at *P* < 0.001.

### 3.3 SYP121^ΔC^ mediates a subset of KAT1 gating transitions

The truncated SNARE SYP121^ΔC^ lacks the C‐terminal transmembrane anchor but retains the essential, channel‐interacting domain and binds with the channel N‐terminus in vitro (Grefen et al., 2015; Grefen, Chen, et al., 2010; Honsbein et al., 2011). We used SYP121^ΔC^ as a tool to dissect the impact on KAT1 gating of SYP121 anchoring and to explore the temporal characteristics of their interaction. Co‐expression in whole oocytes showed that SYP121^ΔC^ altered KAT1 gating but, unlike the full‐length SNARE, the effect was to displace the V_1/2_ to more negative voltages with little effect on ensemble conductance g_max_ (Figures 3a and S2). We observed a near‐maximal effect of SYP121^ΔC^ with roughly 0.4 ng SYP121^ΔC^ per oocyte, or 0.14 μM protein on a cell volume basis (Figure S3), and similar results were obtained with KAT1 in inside–out macropatches on applying 1 μM SYP121^ΔC^ to the cytosolic face of the membrane (Figure S4).

**Figure 3:**
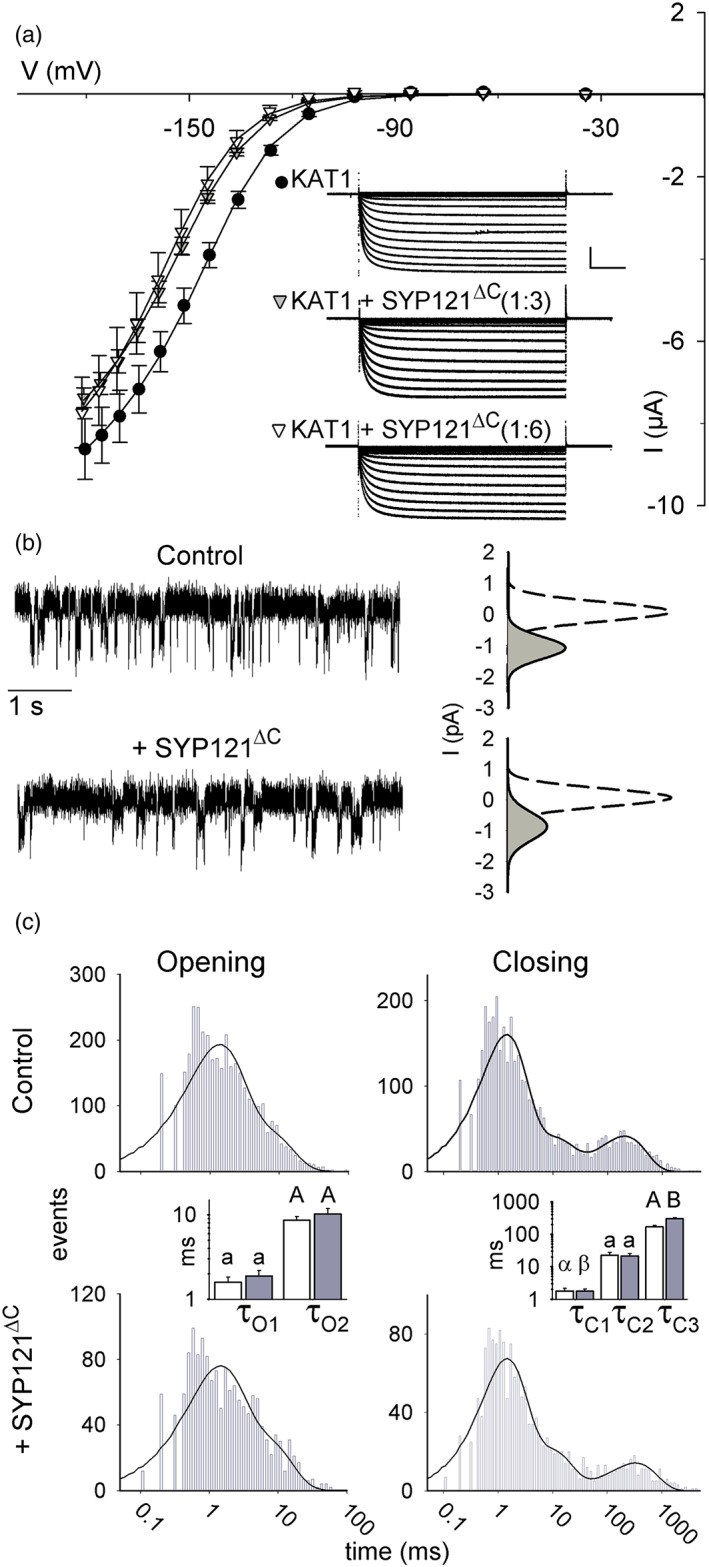
Eliminating the SYP121 membrane anchor recovers a subset only of KAT1 gating transitions. (a) Steady‐state current–voltage curves and representative current traces (*insets*) for KAT1 alone (filled circles) and co‐expressed with the cytosolic SNARE domain, SYP121^ΔC^ (triangles; KAT1: SYP121^ΔC^ ratios: 1:2, grey; 1:6, open). Voltage stepped from −20 mV to voltages between +20 and −180 mV. Solid curves are the result of the joint, least squares fitting to the Boltzmann function of Equation (3). Data are means ±*SE* (*n* ≥ 6). *Insets*: Representative current traces cross‐referenced by symbol. Scale: 2 μA (vertical), 1 s (horizontal). Corresponding Immunoblot analysis shown in Figure S1 and relative conductance curves shown in Figure S3. (b) Representative single‐channel currents recorded at −120 mV from one inside–out patch with KAT1 alone (*above*) and after adding SYP121^ΔC^ to the bath (*below*). Fitted amplitude histograms for the open (*solid lines, grey fill*) and close state (*dashed lines*) taken from the entire 8‐min recording. Open‐state curves are scaled (multiplied) by 4 relative to the closed state for clarity. (c) Frequency histograms of KAT1 open and close durations for KAT1 alone (*above*) and after adding SYP121^ΔC^ to the bath (*below*) plotted on logarithmic scales. Solid lines are fittings to Equation (2) to extract the dwell time constants. Bins below 1 ms (Nyquist limit) are shown but were not included in fittings. Open and closed lifetimes were fitted satisfactorily with sums of two and three exponentials, respectively. Mean dwell time constants ±*SE* (*n* = 6) for open and closed lifetimes are summarized by the bar charts (KAT1, open bars; KAT1+ SYP121^ΔC^, filled bars). In the absence of the SNARE membrane anchor, a significant difference ± SYP121^ΔC^ was observed only for the longest closed dwell time constant, τ_C3_ (see Table 1) [Colour figure can be viewed at http://wileyonlinelibrary.com]

Because the truncated SNARE is soluble, it was possible to add SYP121^ΔC^ directly to the membrane surface during experiments. We recorded single‐channel currents from individual inside–out membrane patches before and after applying 1 μM SYP121^ΔC^ to the cytosolic face of the membrane. Thus, each patch recording included its own internal control. Channel conductance was not affected by SYP121^ΔC^ application and yielded a current amplitude of 1.04 ± 0.16 pA at −120 mV, much as recorded from KAT1 alone (*P* = 0.94). Consistent with the whole‐cell and macropatch recordings, channel P_o_ declined to 3.4 ± 0.2% (*P* = 0.04) after adding SYP121^ΔC^ (Figure 3b). Lifetime analysis (Figure 3c and Table 1) showed that the decrease in P_o_ could be ascribed solely to an increase in the longest‐lived closed state (τ_C3_) in the presence of SYP121^ΔC^ treatment, indicating that the SNARE anchor was important for its full effect on KAT1 gating, especially in promoting the open state (compare with Figure 2; see also Table 1).

**Figure 4:**
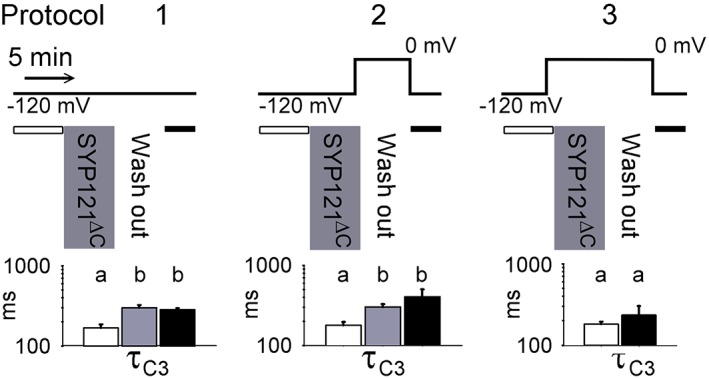
SYP121^ΔC^ interaction with KAT1 is voltage dependent and long‐lived. Dwell time constant analysis for closed lifetimes with KAT1 before (open bars), during additions of SYP121^ΔC^ (grey bars), and after its washout from the bath (black bars). Shown is the largest time constant τ_C3_ used as a proxy for SNARE‐channel interactions (see Figure 3). Protocols above summarize the sequence of treatments and clamp voltages used. Mean dwell time constants ±*SE* (*n* = 4) are indicated below each protocol and in Table 1. Adding SYP121^ΔC^ at −120 mV led to an increase in τ_C3_ that did not recover on washout at −120 mV (*left protocol*) or 0 mV (*centre protocol*). SYP121^ΔC^ had no effect on τ_C3_ when added at 0 mV (*right protocol*) at which the channel is normally closed [Colour figure can be viewed at http://wileyonlinelibrary.com]

We used these characteristics of KAT1 with τ_C3_ as a proxy SYP121^ΔC^ binding and debinding. We reasoned that if the SNARE‐channel interaction was long‐lived, as suggested by the distributed effects of the full‐length SYP121 and the common actions of SYP121 and SYP121^ΔC^ on τ_C3_ (Figure 2 and Table 1), then KAT1 gating should not recover on SYP121^ΔC^ washout. Indeed, following SYP121^ΔC^ additions τ_C3_ failed to return to its pretreatment value, even a prolonged washout of 5 min (Figure 4a).

Membrane voltage drives the channel between conformations that bury much of the VSD in the membrane (the “up” state) when depolarized and that expose it to the cytosol (the “down” state) when hyperpolarized (Latorre et al., 2003; Lefoulon et al., 2014; Palovcak et al., 2014). To test whether depolarized voltages might effect SYP121^ΔC^ release, we repeated these experiments also following washout with the membrane clamped to 0 mV (Figure 4b). The results showed that, following SYP121^ΔC^ additions, τ_C3_ failed to return to its pretreatment value, suggesting that the Qa‐SNARE remains bound to KAT1 even when the channel is closed.

Finally, we tested whether the channel was available for an interaction with SYP121^ΔC^ if the truncated Qa‐SNARE was added, while the channel was in the closed state. SYP121^ΔC^ was added and then washed out with the membrane clamped to 0 mV throughout. In this case, τ_C3_ showed no significant difference before and after treatments with the Qa‐SNARE polypeptide, as if the channel was blind to the SYP121^ΔC^ treatment. This latter finding discounts the trivial explanation of channel rundown. Instead, the most parsimonious interpretation of these experiments is that KAT1 is available for SYP121^ΔC^ interaction in the open channel (VSD down), but not in the closed channel (VSD up) state; and, once bound, the channel and Qa‐SNARE polypeptide remain together in both the open and closed states.

## 4 DISCUSSION

A major action of the Qa‐SNARE protein SYP121 is on the activity of K^+^ channels already situated in the plasma membrane and independent of K^+^ channel traffic (Karnik et al., 2017). Previous work showed that the SYP121 interacts with KAT1 and the closely related KC1 channel subunits in vitro (Honsbein et al., 2009; Honsbein et al., 2011), and binding with KC1 in roots was essential for channel mediated K^+^ uptake in vivo and growth (Honsbein et al., 2009). These and additional studies isolated the binding motifs to the N‐terminal F^9^xRF motif of SYP121 and the RYxxWE motif immediately preceding the first transmembrane α‐helices of the K^+^ channels (Grefen et al., 2015; Grefen, Chen, et al., 2010; Karnik et al., 2017). Although SNARE‐channel binding promotes vesicle traffic as well as channel‐mediated K^+^ influx, how these two, physiologically distinct processes might be temporally coordinated has remained obscure. Indeed, the kinetics of the SNARE‐channel interaction is critical to understanding how channel activity is integrated with the SNARE cycle for vesicle fusion.

Direct access to these kinetics is most readily achieved by monitoring single‐channel gating, which can report on the temporal behaviour of a single, functional channel protein and its conformational transitions between the open and closed states. We have used the gating lifetime distributions of the KAT1 K^+^ channel to explore these conformational transitions and the effects of SNARE interaction in isolation after heterologous expression in *Xenopus* oocytes. Our findings now demonstrate that SYP121 exerts a major influence on the channel, altering its gating by stabilizing the open state of the channel, displacing its voltage‐dependence, and increasing the mean open probability of the channel to promote K^+^ influx. We find that SYP121 action on channel opening depends on SNARE anchoring with the K^+^ channel in the plasma membrane, suggesting that its leverage favours the open state of the channel. Furthermore, we show that SNARE interaction with the channel is long‐lived, maintained in both the open and closed states, implying that the bound channel is likely to be integrated within the SNARE complex throughout its assembly and the much longer process of vesicle fusion.

### 4.1 SYP121 action on KAT1 gating is distributed between state transitions

Co‐expression studies in oocytes and analysis of SYP121 action on heterotetrameric channels assembled of KC1 and AKT1 in the plant had shown that SYP121 selectively displaces the V_1/2_ by as much as +40 mV and enhances the ensemble conductance (Grefen, Chen, et al., 2010; Honsbein et al., 2009). Although we note some quantitative differences, the overall effect of SYP121 on the macroscopic KAT1 current is qualitatively similar. Like the KC1 channel, SYP121 action also depended on the conserved RYxxWE motif at the cytosolic face of the VSD (Grefen et al., 2015; Karnik et al., 2017), and mutation of Trp^62^ in KAT1 was sufficient to eliminate the effects of SYP121 on KAT1 gating (Figure 1a). Heterologous function of the KC1‐AKT1 assembly requires CBL‐dependent kinase expression in oocytes (Honsbein et al., 2009). By contrast, a KAT1 current is recovered in oocytes when the channel subunit is expressed on its own, thus greatly simplifying heterologous analysis.

The VSDs of Kv channels, including that of KAT1, are known to transit between two well‐defined conformational states, an “up” state in which significant portions of the VSD polypeptide are buried within the lipid bilayer, and a “down” state in which much of the inner half of the VSD is exposed to the aqueous phase of the cytosol (Palovcak et al., 2014). The “up” state of KAT1 is associated with the closed channel, whereas the “down” state, which is favoured by membrane hyperpolarization, leads to channel opening (Latorre et al., 2003; Lefoulon et al., 2014). Thus, in principle, in addition to any effects on single‐channel conductance, we may envisage two different mechanisms of action for SNARE binding to alter channel gating. In the first, binding is transient, restricted to periods when the VSD is in the “down” state and largely exposed to the cytosol; in this case, binding may be seen to stabilize this state and prolong the associated channel open lifetime. In the second, binding is long‐lived, occurring in both “up” and “down” states of the VSD, and may be expected to affect channel open as well as closed lifetimes.

**Figure 5:**
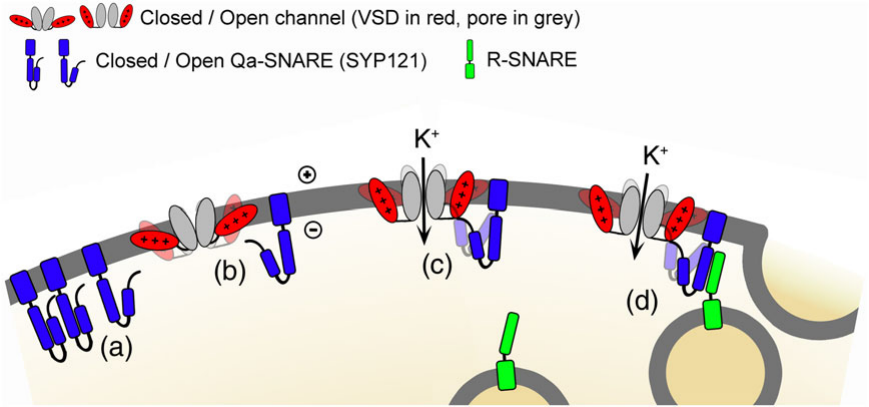
SYP121 binding promotes the open state of KAT1 for K^+^ influx and vesicle fusion. Schematic of the SNARE‐channel interactions deduced from previous analysis of SYP121 characteristics and from KAT1 gating kinetics described here: (a) SYP121 transits between the so‐called closed and open conformations that determine its availability for SNARE complex assembly, the open conformation also favouring channel binding. (b) Channel transitions between open and closed states (down and up conformations of the VSD, shown in red) are shown biased to the closed state, as indicated by the shading. (c) Once bound with SYP121, channel gating is biased to the open state. Transitions to the closed state incorporate a conformational “pull” on SYP121 (shading) and are thereby disfavoured. (d) Bound with the open channel, SYP121 is available for assembly with its cognate R‐SNARE and drives vesicle fusion. The cognate Qbc‐SNARE and other trafficking proteins omitted for clarity

We found that SYP121 had no measurable effect on the single‐channel conductance of KAT1, and its co‐expression yielded no evidence of new kinetic components. Instead, SYP121 action on gating was distributed between three distinct kinetic transitions among those identified for the wild‐type channel (Zei & Aldrich, 1998), consistent with a ΔΔG of approximately −0.4 kcal/mol. Of these, one transition was associated with the open channel and two with the closed channel (Figures 2 and 3 and Table 1). These observations are not easily understood as a consequence of a simple “trapping” of the channel in the open conformation by stabilization of the VSD in the “down” state by the SNARE. Instead, the findings suggest that, once bound, the two proteins remain associated much of the time, the SNARE thereby affecting the lifetimes of both open and closed channel states (Figure 5). Such a temporal association is consistent with the conformational changes of vesicle traffic and fusion, which are comparatively slow (Ales et al., 1999; Fasshauer, Sutton, Brunger, & Jahn, 1998). Furthermore, it suggests that coordinating vesicle traffic with channel gating (Grefen et al., 2015) depends on more subtle conformational changes which are relayed to the SNARE‐bound channel.

### 4.2 SYP121 anchoring to the plasma membrane affects the KAT1 open state

We draw much the same conclusion from studies with the cytosolic domain of the SNARE, SYP121^ΔC^. Using the soluble SYP121^ΔC^ peptide allowed us to challenge KAT1 by adding the SNARE to the bath during recordings, thereby enabling direct comparisons before and after adding the SNARE protein with a single channel. Given that the KAT1 VSD transits between two principal conformations (Latorre et al., 2003; Lefoulon et al., 2014; Palovcak et al., 2014), we anticipated that SYP121^ΔC^ binding might be favoured when KAT1 was held at voltages driving the “down” (open channel) state of the VSD. Indeed, the effects on gating of SYP121^ΔC^, as assayed by the longest closed lifetime τ_C3_, could be circumvented if the SNARE was present only when the voltage clamped to 0 mV to drive KAT1 closed (VSD in the “up” conformation). These findings are consistent with bimolecular fluorescence complementation studies indicating a stronger interaction with the ensemble of open channels incorporating KC1 (Grefen et al., 2015). Furthermore, once SYP121^ΔC^‐mediated gating was altered, subsequent washout both at −120 and at 0 mV, failed to return KAT1 lifetimes to their pretreatment values (Figure 4 and Table 1), at least over the 10‐ to 20‐min periods of these recordings. The observations appear counterintuitive, as the VSD assumes an “up” state conformation that buries much of the VSD in the membrane when KAT1 is closed (Lai et al., 2005; Latorre et al., 2003; Lefoulon et al., 2014; Palovcak et al., 2014). Nonetheless, the SNARE‐binding site, associated with the RYxxWE motif, may remain within the aqueous domain of the cytosol. Regardless, it is clear that the longevity of SNARE action is much greater than even the slowest gating transition that can be resolved for KAT1. The simplest explanation, consistent with the distribution of lifetimes affected by the full‐length SNARE, is that SYP121^ΔC^ binding is facilitated by exposing the binding site and RYxxWE motif in the “down” (open channel) state and, once bound, the SNARE remains associated with the channel for extended periods of time in both “down” and “up” states of the VSD (Figure 5).

These studies also highlight a need for SYP121 anchoring in the membrane, as challenging KAT1 with SYP121^ΔC^ returned a subset only of the kinetic alterations associated with the full‐length SNARE (Figures 3 and S4 and Table 1). SYP121 is normally anchored to the plasma membrane through a single, C‐terminal transmembrane domain, well removed from the channel binding motif located at its cytosolic N‐terminus (Grefen, Chen, et al., 2010; Honsbein et al., 2011). Binding by SYP121^ΔC^, which lacks the C‐terminal membrane anchor, favoured the closed state, displacing the macroscopic dependence of gating to more negative voltages, and it affected only the longest‐lived transition of the closed channel. In principle, the SNARE and channel might interact within the lipid bilayer through the proximity of their respective transmembrane domains (Fletcher, Bowden, & Marrion, 2003; Martens et al., 2000; Martens, Sakamoto, Sullivan, Grobaski, & Tamkun, 2001; Mochida, Sheng, Baker, Kobayashi, & Catterall, 1996). Nonetheless, we interpret the loss of effect on the open state of the channel in the context of membrane anchoring of the SNARE per se, rather than any binding between the SYP121 C‐terminus and the channel for two reasons: (a) split‐ubiquitin assays show that SYP121‐channel binding depends solely on the cytosolic motifs of the two proteins (Grefen et al., 2015; Grefen, Chen, et al., 2010; Honsbein et al., 2011); and (b) SYP121 had no impact on gating of the KAT1^W62A^ mutant, indicating a strict dependence on SNARE binding at this site. Plausibly, then, membrane anchoring of SYP121 at its C‐terminus generates leverage for the SNARE to stabilize the open state of KAT1 but has little effect on the longevity of its closed state.

### 4.3 SYP121 action on KAT1 highlights a functional specificity among Kv channels

We stress that SYP121 action on KAT1, its effects on KC1, is fundamentally different from SNARE binding with Kv channels in animals. The few animal Kv channels that are affected by SNARE binding are associated primarily with the mammalian Syntaxin1A, binding the H3 domain of this SNARE (Leung, Kwan, Ng, Kang, & Gaisano, 2007) which is also required for SNARE complex assembly. Such binding is difficult to reconcile with neurotransmitter secretion. By contrast, both KC1 and KAT1 lack SNARE‐binding sequences homologous to the mammalian Kv channels. Instead, channel binding in the plant depends critically on a linear sequence of residues close to the N‐terminus of SYP121, and the effects on K^+^ uptake by roots and guard cells are straightforward and substantial (Eisenach, Chen, Grefen, & Blatt, 2012; Grefen et al., 2015; Honsbein et al., 2009).

Our findings also highlight more subtle differences between the K^+^ channels that interact with SYP121 in the plant and its consequences for K^+^ flux between tissues. Whereas the channels assembled of KC1 and AKT1 provide a major pathway for K^+^ uptake from the soil, KAT1 expression is primarily foliar, notably in stomata (Jezek & Blatt, 2017), and plays a greater part in K^+^ distribution within the plant. The *syp121* mutation virtually eliminates the K^+^ channel current in the root epidermis (Honsbein et al., 2009), but its effect in guard cells is a partial reduction only in basal K^+^ current, independent of its action in slowing KAT1 recycling to the plasma membrane (Eisenach et al., 2012). These characteristics are evident also in the more modest impact of the SNARE on KAT1 gating and displacement in its voltage‐dependence (Figures 1 and 3). Thus, the comparison shows up unexpected differences in SYP121 actions between the channels, differences that are likely to underpin the macroscopic actions of the Qa‐SNARE in supporting foliar solute accumulation while slowing growth in the whole plant (Geelen et al., 2002; Grefen et al., 2015).

## AUTHOR CONTRIBUTIONS

C. L. carried out the voltage and patch clamp experiments and Western blot studies; C. L. analysed the results with M. R. B.; S. W. and R. K. prepared the SNARE constructs and purified the SNARE proteins; C. L. and M. R. B. wrote the manuscript.

## Supporting information

Figure S1. Tail current analysis from oocytes expressing KAT1 alone and with SYP121.
Fig. S2. Co‐expressing KAT1 with SYP121^ΔC^ displaces KAT1 conductance to more negative voltages.
Fig. S3. Quantifying SYP121^ΔC^ action on KAT1 gating.
Fig. S4. Eliminating the SYP121 membrane anchor displaces the voltage dependence of KAT1 to more negative voltages in macropatches.
Table S1. Exponential components for KAT1 single channel frequency histograms with and without SYP121
