# Supplementary material for: Gating control and K+ uptake by the KAT1 K+ channel leaveraged through membrane anchoring of the trafficking protein SYP121
Source: Plant Cell Environ. 2018 Aug 1;41(11):2668–77. doi: 10.1111/pce.13392 (PMC6220998; doi:10.1111/pce.13392)
Supplement: Supplementary file 1 — Figure S1. Tail current analysis from oocytes expressing KAT1 alone and with SYP121. Fig. S2. Co‐expressing KAT1 with SYP121ΔC displaces KAT1 conductance to more negative voltages. Fig. S3. Quantifying SYP121ΔC action on KAT1 gating. Fig. S4. Eliminating the SYP121 membrane anchor displaces the voltage dependence of KAT1 to more negative voltages in macropatches. Table S1. Exponential components for KAT1 single channel frequency histograms with and without SYP121 [file PCE-41-2668-s001.docx]

Supplemental Information


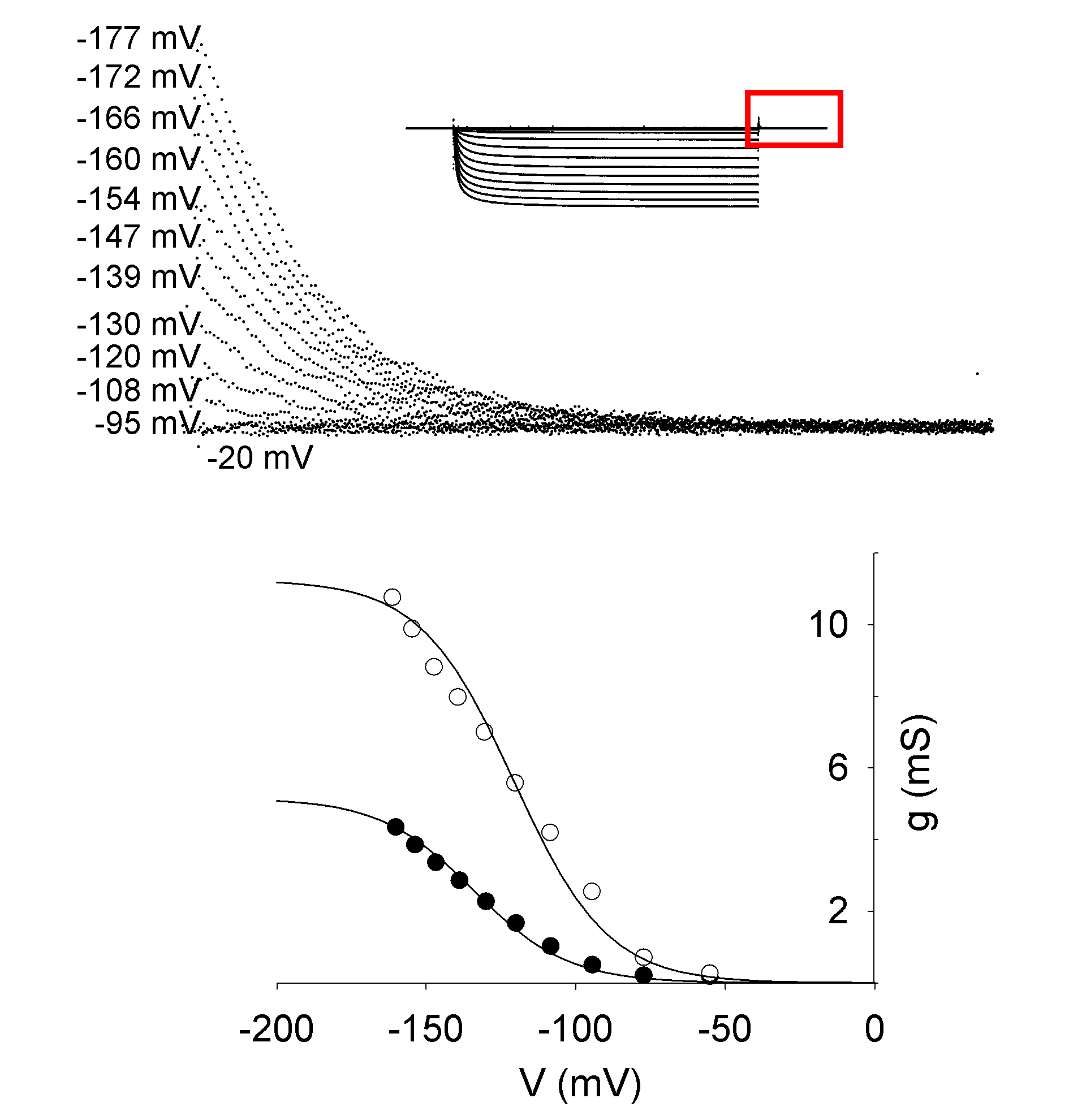


Fig. S1. Tail current analysis from oocytes expressing KAT1 alone and with SYP121.

Current tails (*inset, tails in red rectangle*) recorded at -20 mV are shown expanded as a function of test clamp voltage. The difference in currents recorded at the beginning and end of the -20 mV step were used to estimate the respective conductances and are replotted below for KAT1 alone (filled circles) and with SYP121 (open circles). These conductance values are fitted jointly to a Boltzmann function where

g = g_max_/(1+e^δ(V-V^_1/2_^)F/RT^) [4]

in which V is the test clamp voltage and with the apparent gating charge, δ, held in common between the two sets of data. These data are also shown in Fig. 1B rescaled as relative conductances.


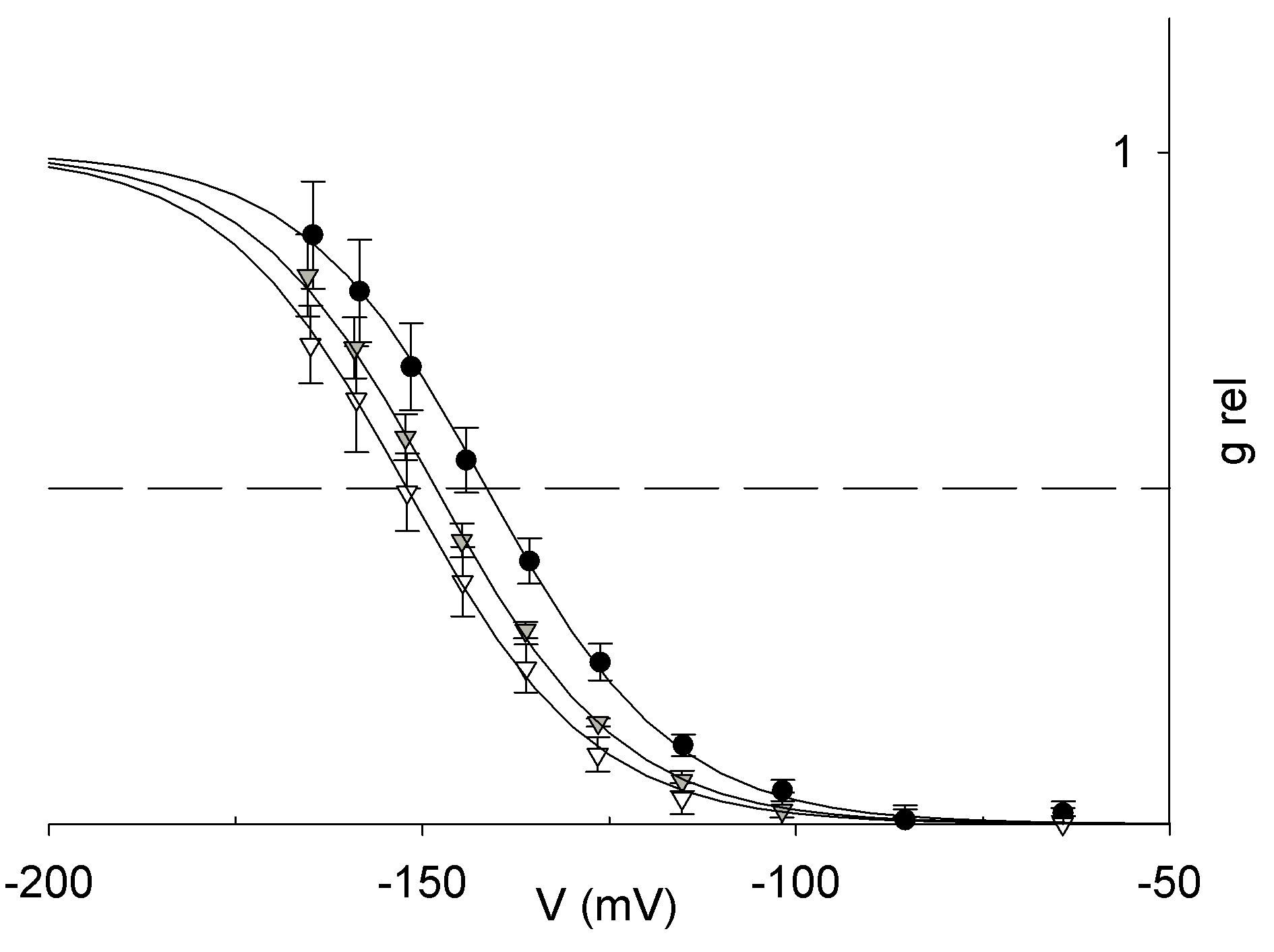
Fig. S2. Co-expressing KAT1 with SYP121^∆C^ displaces KAT1 conductance to more negative voltages.

Relative conductance-voltage curves for KAT1 alone (filled circles) and with SYP121^∆C^ (grey and open triangles) from Figure 3A. Symbols are corresponding relative tail current amplitudes as in Fig. 1. Solid line is the fitting of Boltzmann function derived as g/g_max_ from Eqn [3]. Dashed line marks 0.5 g_max_.


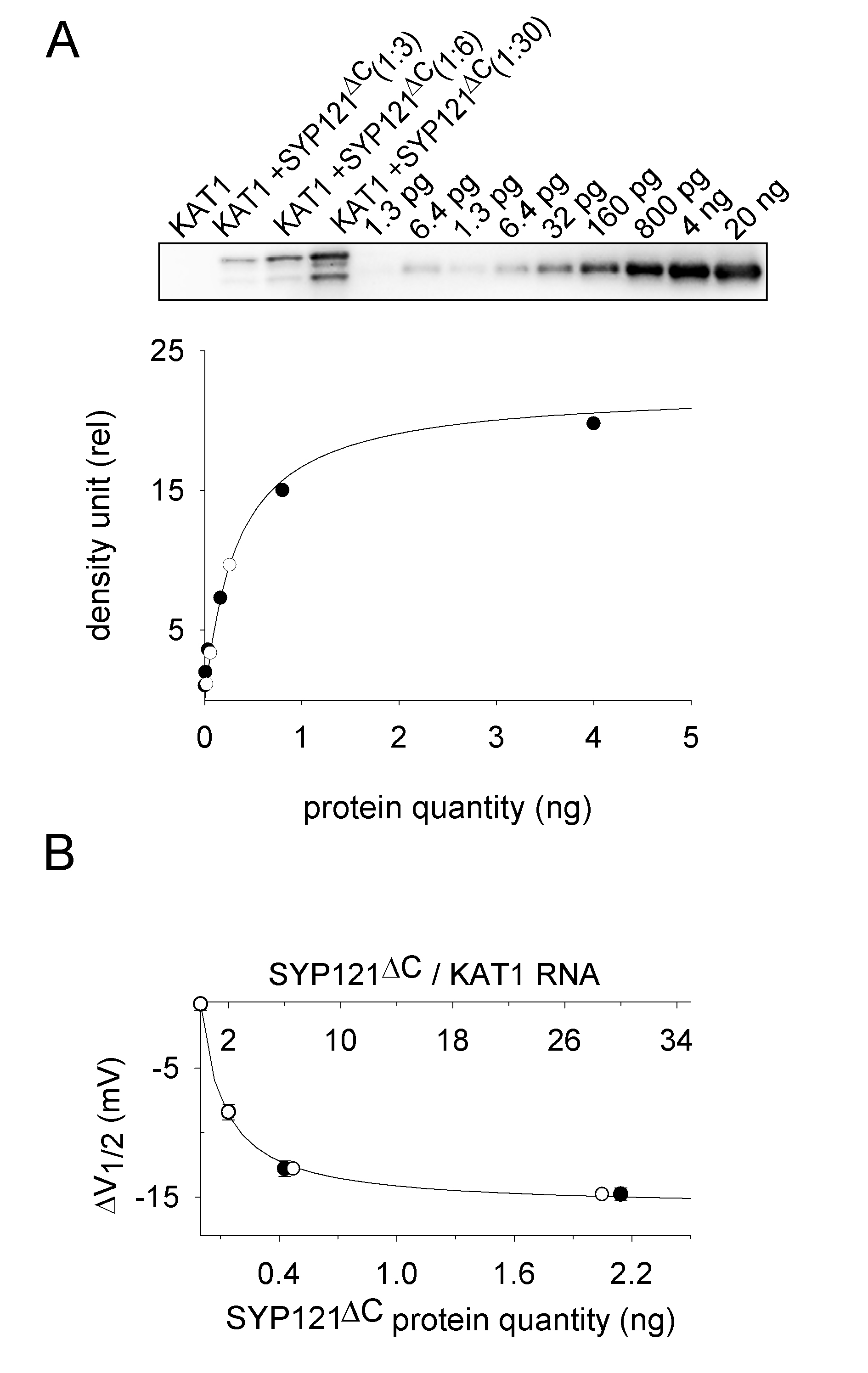


Fig. S3. Quantifying SYP121^∆C^ action on KAT1 gating.

(A) Immunoblot of SYP121^∆C^ from oocytes following recording (see Fig. 3) run together with known amounts of SYP121^∆C^ recombinant protein (*above*). Recombinant protein densities were quantified using Fusion-Capt (Vilber Lourmat, France) are plotted (*below,* filled circles) against protein quantity, and fitted to a simple hyperbolic function to derive a standard curve. SYP121^∆C^ densities from the oocytes are included (open circles) and were used to determine protein quantities after correcting for oocyte volume. Note that SNARE expression in oocytes and in the plant normally yields a band doublet. The slight shift in the SYP121^∆C^ purified following *E.coli* expression probably arises from differences in post-translational modification.

(B) KAT1 gating displacement in V_1/2_ with SYP121^∆C^ plotted against the SYP121^∆C^/KAT1 cRNA ratios (filled circles) and SYP121^∆C^ protein quantity (open circles). Data for the cRNA ratios were fitted to a hyperbolic function (solid curve). The maximum ΔV_1/2_ occurred at -15.8±0.4 mV and the apparent K_1/2_ for SYP121^∆C^ action occurred at a ratio of 1.7±0.2 SYP121^∆C^:KAT1. Similar results were obtained using protein quantities, which indicated near-saturation at 1 ng per oocyte.


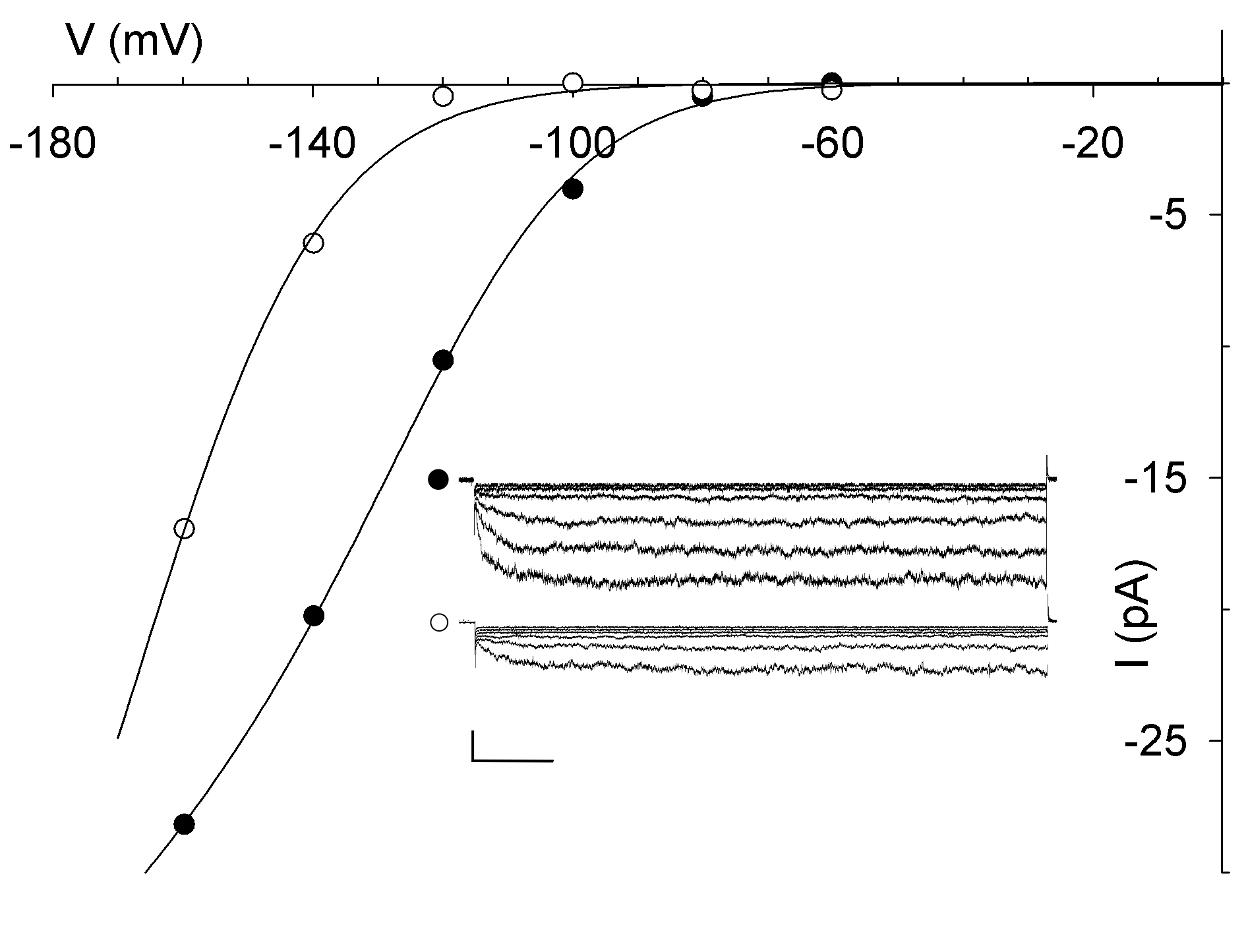
Fig. S4. Eliminating the SYP121 membrane anchor displaces the voltage dependence of KAT1 to more negative voltages in macropatches.

Data from one of five independent experiments shows that adding SYP121^∆C^ to an inside-out macropatch with multiple KAT1 channels leads to a shift in voltage sensitivity of channel gating. Curves are joint, least-squares fitting to the Boltzmann function of Eqn [3] with common values for δ and g_max_ and yielded a -14±2 mV shift in V_1/2_. Inset traces with scale, 10 pA (vertical), 1 s (horizontal).

Table S1. Exponential components for KAT1 single channel frequency histograms with and without SYP121

|  | **Opening component area proportion** | | **Closing component area proportion** | | |
| --- | --- | --- | --- | --- | --- |
|  | a_O1_ | a_O2_ | a_C1_ | a_C2_ | a_C3_ |
| **KAT1** | 73.5 % ± 3.7 | 26.5 % ± 3.7 | 72.0 % ± 1.2 | 13.8 % ± 4.5 | 14.2 % ± 3.6 |
| **KAT1 + SYP121** | 80.4 % ± 7.5 | 19.6 % ± 7.5 | 73.1 % ± 4.4 | 12.9 % ± 3.7 | 14.0 % ± 1.1 |

Exponential component areas from frequency histogram fittings of Fig. 2B as described in the text. Values are reported as percentages of the total area under each curve ±SE. There are no significant differences (P<0.05) between KAT1 expressed in oocytes with or without SYP121.
